# Architecture Effect on Network Phase Formation from Controlled Self-Assembly of High‑χ Block Copolymers

**DOI:** 10.1021/acs.macromol.5c02152

**Published:** 2025-11-17

**Authors:** Cheng-Yen Chang, Gkreti-Maria Manesi, Yun-Hao Chen, Yu-Jie Tsai, Hsing-Yu Su, Apostolos Avgeropoulos, Rong-Ming Ho

**Affiliations:** † Department of Chemical Engineering, 34881 National Tsing Hua University No. 101, Section 2, Kuang-Fu Road, Hsinchu, Taiwan 30013, R.O.C.; ‡ Department of Materials Science Engineering, 37796 University of Ioannina, University Campus, Ioannina 45110, Greece

## Abstract

This work aims to examine the architecture effect on the self-assembly of high-χ block copolymers (BCPs), polystyrene-*block*-polydimethylsiloxane (PS-*b*-PDMS). Lamellae-forming diblock and star BCPs with three and six arms were synthesized for self-assembly. A variety of self-assembled phases, especially network phases, can be formed by tuning the solvent evaporation rate for solution casting using PS-selective solvents (referred to as controlled self-assembly). In contrast to diblocks with linear architecture, the formation of network phases from star BCPs can be easily acquired via controlled self-assembly. It is possible to enrich the topological features of the forming network phases, giving double gyroid and Frank–Kasper-like network phases, with alternating three- and four-strut nodes, as well as a double diamond phase due to the alleviation of packing frustration from network formation. As a result, the architecture effect on the phase behaviors of high-χ BCPs via controlled self-assembly gives easy access to acquire a variety of network phases.

## Graphical abstract

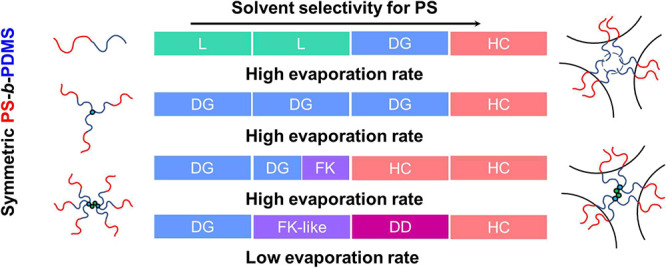

## Introduction

Self-assembly of block copolymers (BCPs) is a spontaneous microphase separation among the chemically incompatible blocks, giving the formation of a variety of ordered phases with one-, two-, and three-dimensional nanostructures.^1–3^ Among them, the network phases are the most fascinating ones due to their bicontinuous frameworks. The examples are photonic crystals on butterfly wings and the calcite skeleton beneath a starfish skin, inspiring the applications in optical and mechanical properties due to their deliberate structuring.^4–12^ Unlike the top-down techniques, the self-assembly of BCPs is a bottom-up method capable of achieving various mesoscale structures. Research studies have been extensively concentrating on network phases, such as gyroid and diamond, with a triply periodic minimal surface.^13–15^ The interfacial curvatures among the microdomains of constituted blocks are the minimum free energy state, a balance between enthalpy and entropy, resulting in an intermediate between a constant mean curvature and a constant mean thickness model based on self-consistent field theory (SCFT).^16^ Thus, the junction of the struts (i.e., the node) expands with the increase of strut number, leading to a larger volume required for filling of matters at the nodes, which is referred to as packing frustration due to the overstretching of polymer chains.^17,18^ As a result, there are strict requirements for the polymer chains, where only dedicatedly designed degree of polymerization and composition ratios can stabilize the structure, confining a narrow window for network phases in the phase diagram of BCPs;^19^ to obtain network phases, precise synthesis is achievable but still difficult to control the volume fraction and the degree of segregation strength (χ*N*) that are rightly located in the narrow window. As alternatives, various approaches are proposed to control the volume fractions of the constitutive components to fulfill the criteria for network phase formation. Common approaches are binary or ternary blending with homopolymers or BCPs so that those additives and the host BCPs are able to fill the large space in nodes, which stabilizes the network structures.^20–24^ In principle, uniform distributions of polymer chains within the space are the keys to solving packing frustration. Apart from those, appropriate molecular design alters the properties of BCPs to adjust their molecular architectures.^25,26^ For instance, end-group chemistry imposes the strong interaction among end-groups on each BCP chain end, giving association of those at the nodes, which mitigates the high packing frustration.^18,27–31^ Another way to acquire network phases is by connecting chain ends with multifunctional coupling agents to form star-shaped copolymers. The molecular architecture reduces the entropic penalty on self-assembly and thus shifts the phase diagram, which is predicted by SCFT calculations, suggesting that network phases become more stable in the self-assembly of star BCPs due to the architecture effect.^32,33^ Li's group further investigated the effect of conformational asymmetry created by the nonlinear BCPs, especially for the branched architectures, on the self-assembled phase behaviors and the origin of complex phases.^34–36^ Besides the approaches mentioned above, another straightforward method to acquire network phases is to use selective solvents to swell particular blocks at a specific degree, giving the feasibility to acquire aimed network phases via phase transitions from BCP solutions.^37–39^ However, the low χ value of the extensively studied system, polystyrene-*block*-polyisoprene (PS-*b*-PI), might cause lower segregation strength, resulting in challenges in self-assembly and ordering of phases. In contrast, by taking advantage of a high-χ system in the literature studies, it is favorable for microphase separation, which benefits the stability and the ordering of the self-assembled phases.^40^ Therefore, by using high-χ BCPs for self-assembly from solution with the use of a solvent possessing specific solvent selectivity between their two blocks, diverse self-assembled phases with network textures can be acquired from a single-composition symmetric diblock.^41^ This method shows the simplicity of tuning the selectivity of solvents to obtain network phases. Furthermore, by controlling the evaporation rate of selective solvents, distinct network phases with higher packing frustration can be kinetically trapped by tuning the evaporation rate.^42^ As a result, various self-assembled phases can be acquired from a high-χ BCP solution for solution casting through order–order transitions, followed by vitrification during evaporation, which is referred to as controlled self-assembly.

Herein, this work aims to examine the architecture effect on controlled self-assembly of high-χ BCPs, PS-*b*-PDMS. In contrast to linear diblocks, the star-shaped PS-*b*-PDMS with jointed PDMS junctions can alleviate the entropic penalty for the formation of complex network phases. During evaporation, BCPs go through a series of order–order transitions in the solution state and finally can be vitrified at a particular point, which is kinetically controlled by the evaporation rate of the selective solvent. With that, it is possible to give the formation of network phases, including gyroid, diamond, and even a distinct phase referred to as a Frank–Kasper-like (FK-like) network phase. Accordingly, the alternation of the molecular architecture of PS-*b*-PDMS shows great potential to enrich the forming network phases from controlled self-assembly. As a result, by taking advantage of the architecture effect on controlled self-assembly of high-χ BCPs, it is feasible to give the formation of complex network phases through appropriate selection of solvent for solution casting under optimized processing conditions for nearly symmetric BCPs.

## Experimental Section

### Materials

The diblock copolymer (PS-*b*-PDMS) (*M*_n_^PS^ = 15,700 g/mol, *M*_n_^PDMS^ = 11,100 g/mol, *Đ* = 1.03) and the six-arm star BCP ((PS-*b*-PDMS)_6_) with the volume fraction of PDMS, *f*_PDMS_^v^ = 0.42, were used in previous study and in this work.^43,44^ The syntheses of diblocks started from polymerization of styrene using *sec*-Buli as an initiator in a nonpolar environment (benzene), and hexamethylcyclotrisiloxane (D_3_) was introduced afterward, followed by the addition of a polar solvent (tetrahydrofuran), in order to propagate the polymerization for PS-*b*-PDMS^(−)^ Li^(+)^. Two different coupling agents were used to synthesize PS-*b*-PDMS to achieve the three-arm and six-arm star BCPs with identical chain length for each arm (see references for more details).^43,45^ All samples are listed in Table 1.

**Table 1:** Characterization of Synthesized (PS-*b*-PDMS)_*n*_ (*n* = 1, 3, and 6)

samples	${{\overset{-}{M}}_{n}}^{\mathrm{PS}}$ ^a^ (g/mol)	${{\overset{-}{M}}_{n}}^{\mathrm{PDMS}}$ ^a^ (g/mol)	*Đ* ^b^	${f_{\mathrm{PDM}S}}^{v}$ ^c^
PS-*b*-PDMS	15,700	11,100	1.03	0.42
(PS-*b*-PDMS)_3_	38,400	30,900	1.03	0.46
(PS-*b*-PDMS)_6_	94,200	66,600	1.05	0.42

a Total number-average molecular weights corresponding to two individual blocks (PS and PDMS) determined by vapor pressure and/or membrane osmometry (VPO and/or MO).

b Polydispersity determined by size exclusion chromatography.

c Volume fraction of PDMS as calculated from proton nuclear magnetic resonance spectroscopy (^1^H NMR) using densities of PS and PDMS (ρ_PS_ = 1.04 g/cm^3^, ρ_PDMS_ = 0.97 g/cm^3^).

### Sample Preparation for Solution Casting

All the bulk samples were prepared by solution casting with various solvents, including cyclohexane, chloroform, toluene, dichloromethane, and chlorobenzene, under two evaporation rates for controlled self-assembly of all PS-*b*-PDMS synthesized. The BCP solution was prepared by dissolving 50 mg of PS-*b*-PDMS in stoichiometric amounts of solvent at a fixed concentration of 10 wt % in a cylindrical glass container. Fast evaporation rate was carried out at 0.1 mL/h. The whole procedure of solution casting takes approximately 3 h at room temperature. On the other hand, the containers were sealed with caps with open holes to reduce the evaporation rate to 0.1 mL/day, which took 3 days for the solvent to dry out at room temperature. After drying the bulk samples at ambient conditions, the bulk samples were soaked in liquid nitrogen and detached from the glass to prevent distortion.

### Self-Assembled Morphology Characterization

Ultrathin microsections of solution-cast PS-*b*-PDMS, including diblock and star-block (thickness lower than 100 nm), were prepared by a Leica UC6 Ultramicrotome at −160 °C. Without using any staining agent, bright-field TEM imaging can be directly acquired from the microsections with intrinsic mass contrast from silicon-containing microdomains (PDMS) to hydrocarbon microdomains (PS). Bright-field TEM observations were carried out on a JEOL-2100 transmission electron microscope operated at an accelerating voltage of 200 kV or on a Hitachi HT7700 TEM operated at an accelerating voltage of 100 kV.

### Small-Angle X-ray Scattering Experiments

The small-angle X-ray scattering (SAXS) experiments using synchrotron beam sources were completed at beamline TLS 23A and beamline TPS 13A of the National Synchrotron Radiation Research Center. The incident X-ray beam (λ = 1.24 Å) at TLS 23A was vertically focused through a mirror and a germanium (111) double-crystal monochromator monochromated to an energy of 10 keV. The beam size was approximately 200 μm in diameter. The feature size of the beam stop (a round tantalum disk) was 4 mm in diameter. All of the two-dimensional (2D) SAXS patterns were collected by an MAR CCD X-ray detector (MAR USA) and azimuthally integrated to one-dimensional (1D) SAXS profiles. The incident X-ray beam (λ = 0.83 Å) at TPS 13A to the energy of 15 keV was aligned through a setup similar to that of TLS 23A. A microbeam with dimensions (133 μm) was used for exposure to samples. All the 2D SAXS patterns were collected by an Eiger X 9M CCD at a distance to 10 m for the *q* region (0.015 nm^–1^ < *q* < 1.5 nm^–1^) and an Eiger X 1M CCD at a distance to 0.84 m for the *q* region for wide-angle scattering (*q* < 20 nm^–1^). One-dimensional SWAXS profiles can be azimuthally integrated and merged from the collected SAXS patterns from both detectors.

## Results and Discussion

### Characterization of Lamellae-Forming PS-*b*-PDMS

Table 1 shows the lamellae (L)-forming PS-*b*-PDMS BCPs synthesized, including diblock and star-block PS-*b*-PDMS, with the volume fractions of PDMS (*f*_PDMS_^v^) ranging from 0.42 to 0.46 for controlled self-assembly (see the Supporting Information and Figures S1–S6 for details with respect to synthetic routes, proton nuclear magnetic resonance (^1^H NMR) spectra, and gel permeation chromatography (GPC) results of the synthesized BCPs). To examine the equilibrium phase of the synthesized samples, a neutral solvent for PS and PDMS, cyclohexane, was used for solution casting. The SAXS profiles of all samples in Figures S7A suggest the formation of the L phase, at which the reflections occur at the relative *q* values of 1:2:3:4. Alternating dark and bright stripes were observed under TEM, as shown in Figure S7B–D; the dark stripes are referred to as silicon-containing microdomains (PDMS) due to the mass contrast, whereas the PS microdomains appear bright, further evidencing the formation of an L phase after solution casting by using a neutral solvent for all the synthesized samples. Furthermore, the cast samples above all appear as the L phase, regardless of the evaporation rate during solution casting.

### Architecture Effect on Self-Assembly of Star-Block PS-*b*-PDMS

By using PS-selective solvents for solution casting of lamellae-forming PS-*b*-PDMS to lower the effective volume fraction of PDMS (*f*_PDMS_^v,eff^), metastable network phases, such as gyroid and diamond, can be acquired through order–order transitions, followed by vitrification during solvent evaporation (i.e., controlled self-assembly). Chloroform, toluene, dichloromethane, and chlorobenzene were used as PS-selective solvents for the controlled self-assembly (see Figure S8 and the Supporting Information for detailed description of determination of solvent selectivity to the constituted blocks in PS-*b*-PDMS and the estimation of *f*_PDMS_^v,eff^). As shown in Figure 1A (blue and red lines), the SAXS profiles for the diblock, PS-*b*-PDMS, cast by using chloroform and toluene (referred to weakly PS-selective solvent), respectively, showing reflections occurred at the relative *q* values reveal that the volumetric compositions of the constituted blocks in the diblock remain in the composition window for the formation of an L phase after vitrification. The SAXS profile (purple line in Figure 1A) changes when dichloromethane (referred to as the intermediate PS-selective solvent) was used for casting; the reflections at the relative *q* values of √6:√8:√14:√20:√26:√32:√40:√50 suggest the formation of a double gyroid (DG) phase. Figure 1D shows the corresponding TEM micrograph with grating-like contours, which is consistent with the [211]_DG_ projection of DG, further confirming the kinetically trapped phase with the reduction of *f*_PDMS_^v,eff^ after vitrification. With the use of a highly PS-selective solvent, chlorobenzene, for the controlled self-assembly, a hexagonally packed cylinder (HC) phase is formed, as evidenced by the SAXS profile, where the reflections are at the relative *q* values of 1:√4:√7:√9:√16 (brown line in Figure 1A). Consistently, the hexagonally packed dark microdomains referred to PDMS can be observed under TEM (Figure 1E), suggesting that the use of chlorobenzene greatly reduces the *f*_PDMS_^v,eff^ to highly asymmetric composition. Accordingly, it is possible to kinetically capture the self-assembled morphologies at local minimum Gibbs free energy states from the PS-*b*-PDMS solution after vitrification of an ongoing phase transition during evaporation. By contrast, as shown in Figure 2A, when weakly PS-selective solvents (chloroform and toluene) were used for controlled self-assembly of the three-arm star BCP, (PS-*b*-PDMS)_3_, multiple reflections at the relative *q* values of √6:√8:√14:√40:√50 and √6:√8:√14:√22:√38:√50 (blue and red lines, respectively) were found, where the one at √6 shows the highest intensity and is referred to the primary peak; the profiles suggest the formation of DG. Note that the appearance of √2 and √4 is attributed to the deformation of the self-assembled morphology.^46–48^ A similar SAXS profile (purple line in Figure 2A) was found while using an intermediate PS-selective solvent (dichloromethane), indicating that a DG phase can be captured. The characteristic [110]_DG_ projection of the DG phase was observed under TEM, as shown in Figure 2B–D. In contrast to the diblock copolymer, there is a greater opportunity to capture the DG phase, inferring that the architecture effect on BCPs is feasible to solve the packing frustration difficulty in the network phase. With the use of the highly PS-selective solvent (chlorobenzene), a SAXS profile showing reflections corresponding to the HC phase was obtained, in agreement with the results of its diblock precursor as discussed above. As shown in Figure 2E, dark columns (PDMS microdomains) are well aligned and arranged into a hexagonal packing in the PS matrix, remarking the significant reduction in *f*_PDMS_^v,eff^ that shifts toward asymmetric composition. Further increasing the arm number of star BCPs to 6, the DG phase can be acquired by using chloroform for solution casting of a six-arm star BCP (PS-*b*-PDMS)_6_, as shown in Figure 3A (blue line). Figure[Fig fig3]B displays the projection of DG along [210]_DG_ under TEM. Interestingly, after solution casting by toluene, mixed phases of a unique network and DG phases were found, which contribute to the multiple reflections in the SAXS profile (red line in Figure 3A). The reflection peaks marked by blue rods represent the reflections from the phase with the space group *Pm*3̅*n* (FK), which is considered as the FK-like network phase composed of alternating three- and four-strut building units,^44^ and those marked by black rods represent the reflection from a DG. The [100] projection of the FK-like network phase can be observed under TEM, as shown in the upper right part of Figure 3C. Also, the [111] projection of DG appears in the lower left part of Figure 3C, further confirming the formation of mixed phases of the FK-like network phase and DG. According to the report from Ho et al.,^44^ the packing frustration of the FK-like network phase is considered to be larger than that of DG due to the difference in coordination numbers of the building units, revealing that the six-arm star BCP has the capability to overcome the high packing frustration. After solution casting by dichloromethane, reflections (purple line in Figure 3A) at the relative *q* values of 1:√3:√4:√7:√9:√12 suggest the formation of an HC phase. Round PDMS microdomains were observed under TEM, as shown in Figure 3D, which is in line with the SAXS profile. With the use of chlorobenzene, the collected SAXS profile (brown line in Figure 3A) suggests the formation of an HC phase, which was validated by the TEM image, as shown in Figure 3E. Conclusively, as the architecture effect was introduced (i.e., increases on arm numbers), it is more likely to enrich the phase behaviors, especially the formation of network phases, from the controlled self-assembly of PS-*b*-PDMS. Specifically, the connection at the center of the PDMS core effectively reduces the entropic penalty, while the polymer chains are stretched to reach the node of structure for the formation of network phases from the BCP solution via the controlled self-assembly.

**Figure 1:**
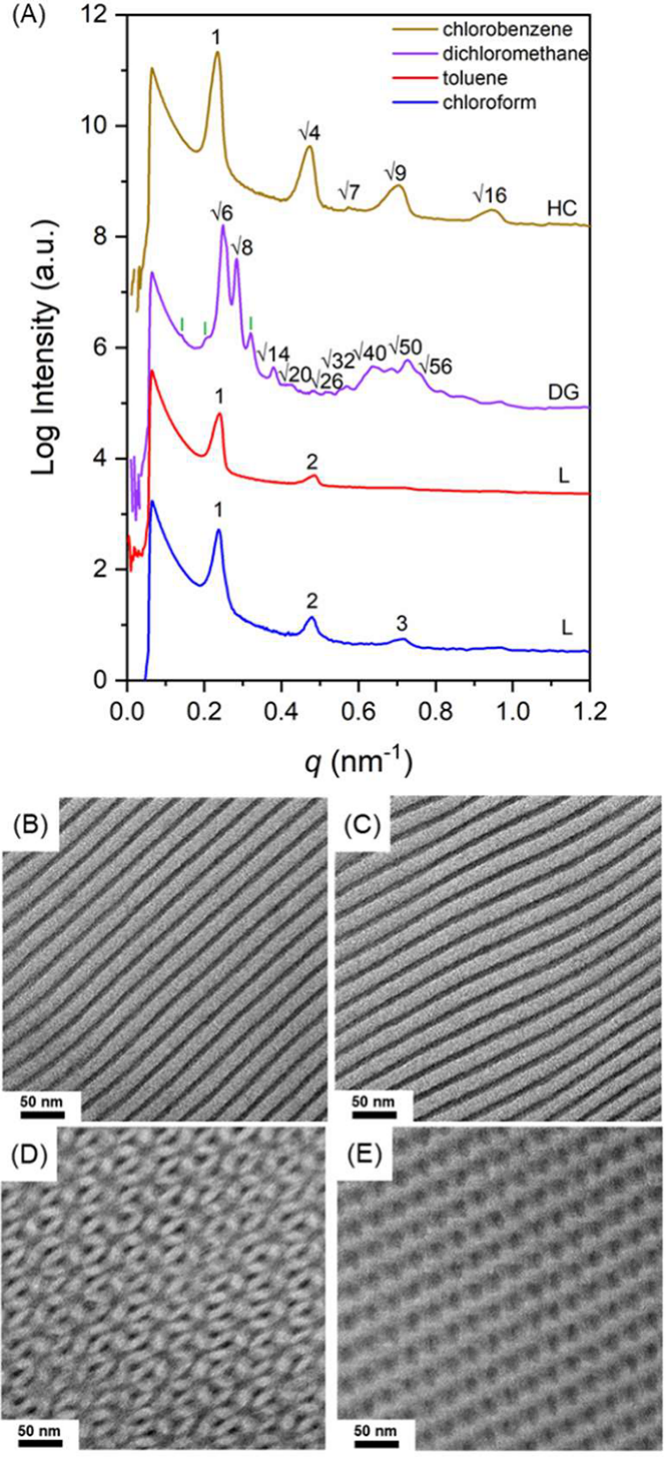
(A) One-dimensional SAXS profiles; (B–E) TEM micrographs of PS-*b*-PDMS from solution casting using chloroform, toluene, dichloromethane, and chlorobenzene at a fast evaporation rate of 0.1 mL/h.

**Figure 2:**
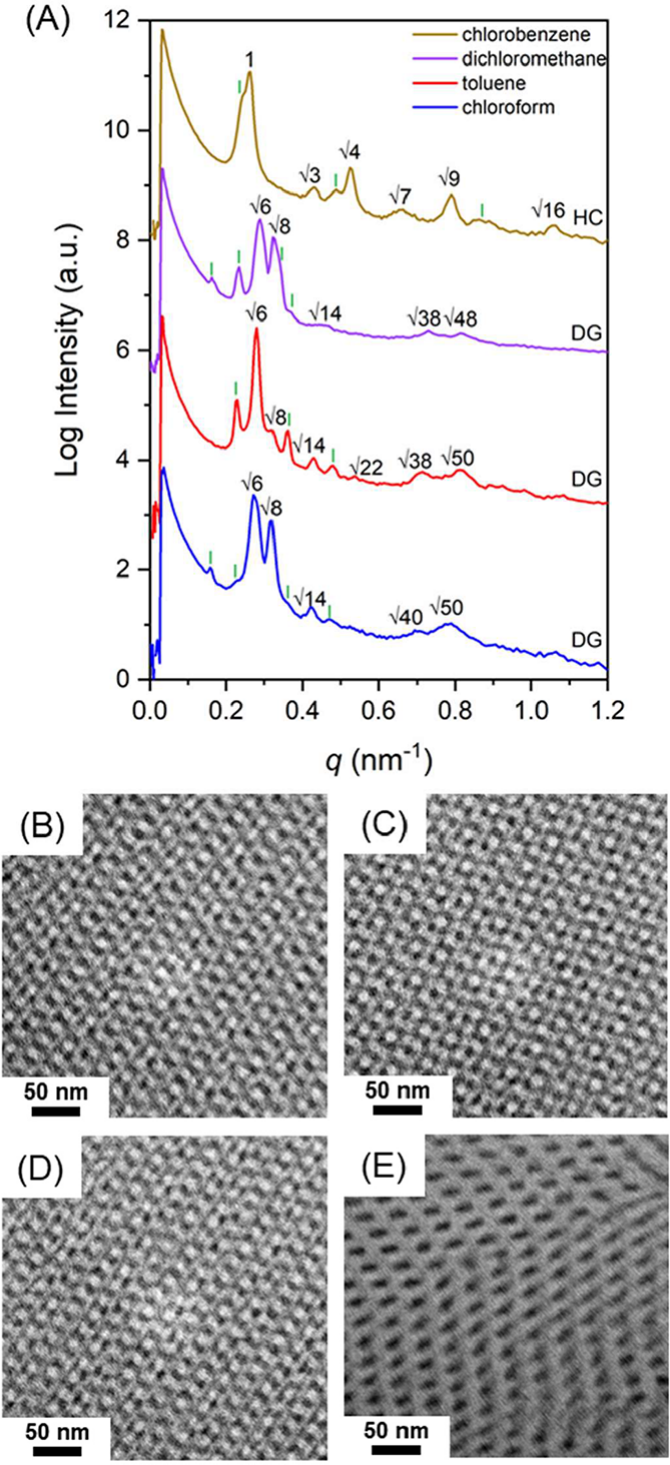
(A) One-dimensional SAXS profiles and (B–E) TEM micrographs of (PS-*b*-PDMS)_3_ prepared from solution casting using chloroform, toluene, dichloromethane, and chlorobenzene at a fast evaporation rate of 0.1 mL/h.

**Figure 3:**
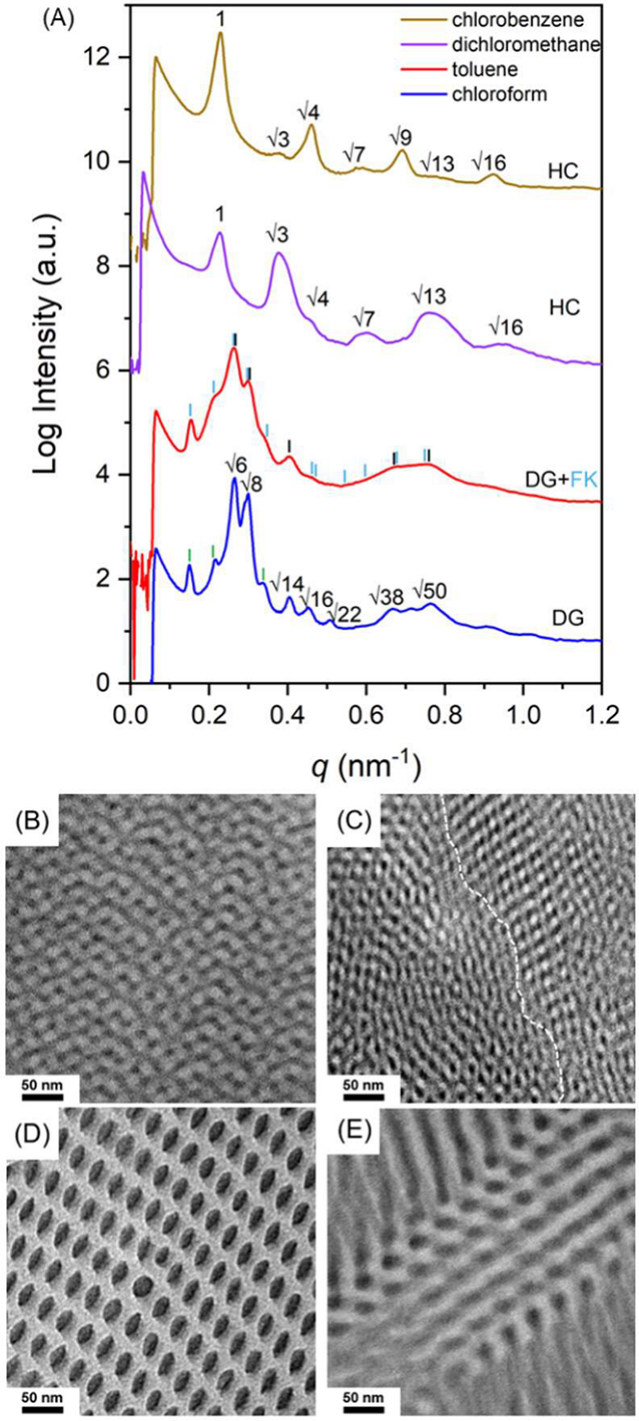
(A) One-dimensional SAXS profiles and (B–E) TEM micrographs of (PS-*b*-PDMS)_6_ prepared from solution casting using chloroform, toluene, dichloromethane, and chlorobenzene at a fast evaporation rate of 0.1 mL/h. The white dashed line in panel (C) indicates the phase boundary between the DG (left) and FK-like network phase (right).

### Kinetic Effect on Controlled Self-Assembly of PS-*b*-PDMS

As demonstrated above, the controlled self-assembly provides an alternative pathway to acquire metastable phases from the PS-*b*-PDMS solution with the use of PS-selective solvent via sequential order–order transitions before vitrification of the PS matrix during evaporation for solution casting of the easy synthesis of lamellae-forming BCPs. It is reasonable to expect that, by lowering the evaporation rate, there will be longer extensions for the development of order–order transitions before vitrification due to the extended time for the rearrangement of the BCP chains, getting higher probability to go through multiple local minimum Gibbs free energy states, and that essentially brings the complexity to the self-assembled phase behaviors, which enriches the formation of different morphologies after vitrification. By setting the evaporation rate to 0.1 mL/day (referred to slow evaporation rate) as compared to 0.1 mL/h used above, as shown in Figure S9, there are similar results for the diblock and three-arm star BCPs (Figure S10). In contrast, short evaporation may result in the formation of disordered morphologies due to the unorganized microphase separation of the BCP chains.^42,50^ Figure S9B,E shows the corresponding TEM micrographs of the self-assembled phases according to the solvent that was used for casting. Clear projections of the DG phase along the [110] and [321] directions and hexagonally packed patterns of PDMS microdomains from the HC phase, respectively, were captured. Most interestingly, in the case of a six-arm star BCP, a dramatic variation for the self-assembled results can be found by lowering the evaporation rate; the distinct FK-like network phase can be isolated. Various network phases, including the DG phase, FK-like network phase, and DD phase, can be obtained (Figure S11).^44^ Those results further evidence the suggested mechanisms of forming metastable phases as the outcomes of vitrification of a specific state within a series of order–order transitions during controlled self-assembly. The increase of arm numbers reduces the entropy of molecular conformations that influence the kinetics of self-assembly and thus brings the diversity for order–order transition by decelerating the evaporation rate and also confines free spaces for rearrangement of chains to fix the packing frustration problem.^51–53^ Accordingly, it is feasible to acquire network phases with higher theoretical packing frustration, such as the DD phase, and to exclusively trap the FK-like network phase during multistep order–order transitions from highly frustrated structures to the equilibrium state.

## Conclusions

In this study, the architecture effect on controlled self-assembly of PS-*b*-PDMS with nearly symmetric compositions, including diblocks and star-blocks BCPs, was examined. The architecture effect results in distinguishable differences in self-assembled phase behaviors between diblocks and star-blocks, suggesting an alternative approach to capture the network phases through controlled self-assembly. Furthermore, for diblocks, as illustrated in Figure 4A, strict requirements for the stretching chains (i.e., entropic penalty) are demanded to fill the space in nodes of the forming network uniformly without creating voids, even at the swollen state when polymer chains are stretched, and the presence of solvent molecules may reduce the segregation strength of the strongly segregated system.^54^ Specifically, the gradual radial distribution might not be negligible so that it can influence the packing frustration of the forming phase.^35^ Ideally, for morphologies with constant thickness, such as spheres, cylinders, and lamellae, the distribution of the minor blocks is even, and thus, the polymer chains are stretched uniformly.^55^ In contrast, the gyroid curvature creates fluctuations in the thickness of struts where the minor blocks fill and, therefore, results in a higher degree of packing frustration. Star BCPs with higher arm numbers were proven to be capable of amplifying the architecture effect due to the multiple chemically bonded polymer chains on a fixed junction, as shown in Figure 4B,C. The star-shaped architecture balances the stretching stress and creates higher conformational asymmetry because of the entropy loss caused by confined chain stretching in the core (i.e., PDMS block), preferentially contributing to the spontaneous curvature.^32,34,36^ To be specific, the three-arm star BCPs are able to overcome the packing frustration when using the same solvents (chloroform and toluene), as summarized in Figure 5. As compared to a three-arm star BCP, a six-arm star BCP gives more diversity for network phase formation, at which network phases with higher packing frustration, such as DD and FK-like network phases (DCM and toluene, respectively), other than the DG phase, can be formed; theoretically, the six-arm-star-shaped molecular architecture creates a higher degree of conformational asymmetry, consequently giving the spontaneous curvature to the gradual radial distribution of the constituted blocks. Moreover, different evaporation rates for solution casting with the use of selective solvent were set to study the kinetic factors during controlled self-assembly: a fast evaporation rate results in shorter time for the ongoing order–order transitions based on the variation of *f*_PDMS_^v,eff^ before vitrification of the glassy matrix that commonly leads to the formation of metastable phases in the early stages and with a lower degree of ordering. As the evaporation rate decelerates, the ongoing order–order transitions to network phases result in the formation of network phases with a higher degree of ordering; under some conditions, a single phase was isolated from two-phase mixtures. Consequently, the architecture effect demonstrates the feasibility of enriching phase behaviors by tuning both thermodynamic and kinetic effects. Accordingly, it is possible to capture the desired metastable network phases by controlled self-assembly of star-block PS-*b*-PDMS via controlled self-assembly.

**Figure 4:**
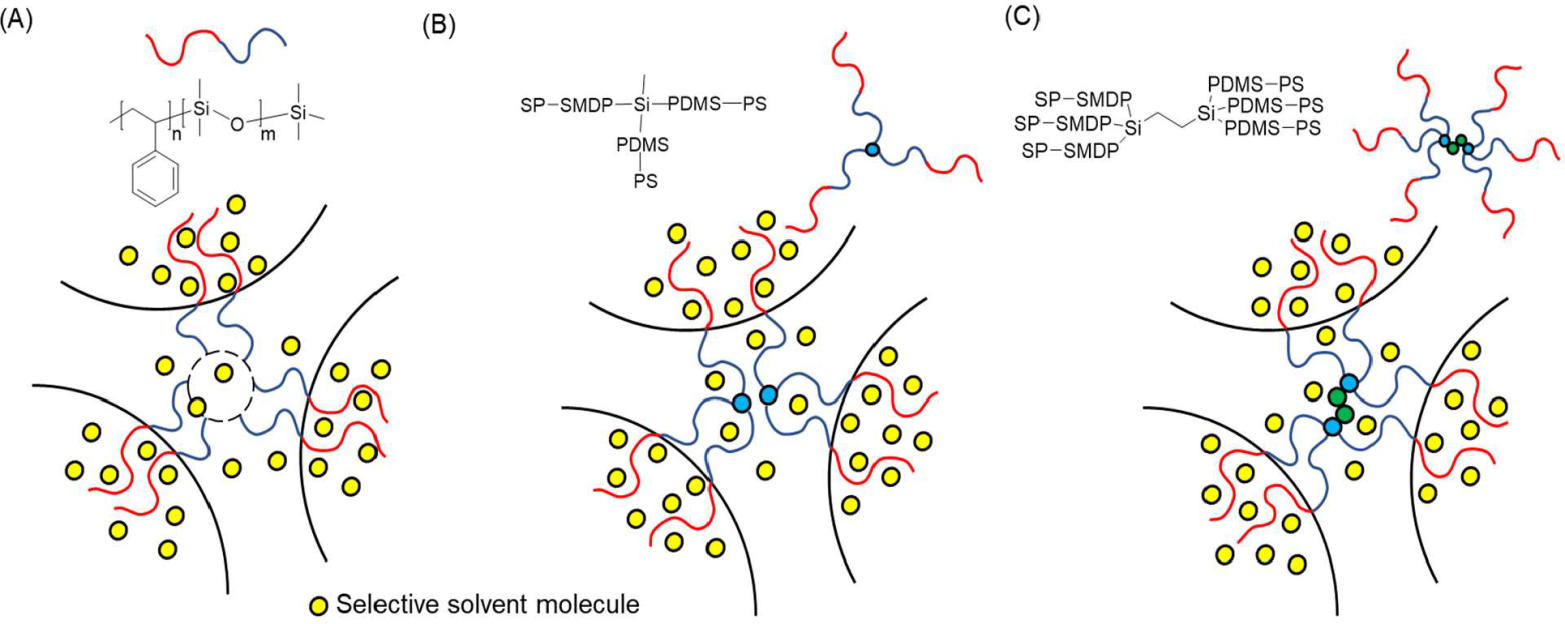
Schematic illustration of the architecture effect on the controlled self-assembled BCPs of (A) diblock, (B) three-arm, and (C) six-arm star-block PS-*b*-PDMS.

**Figure 5:**
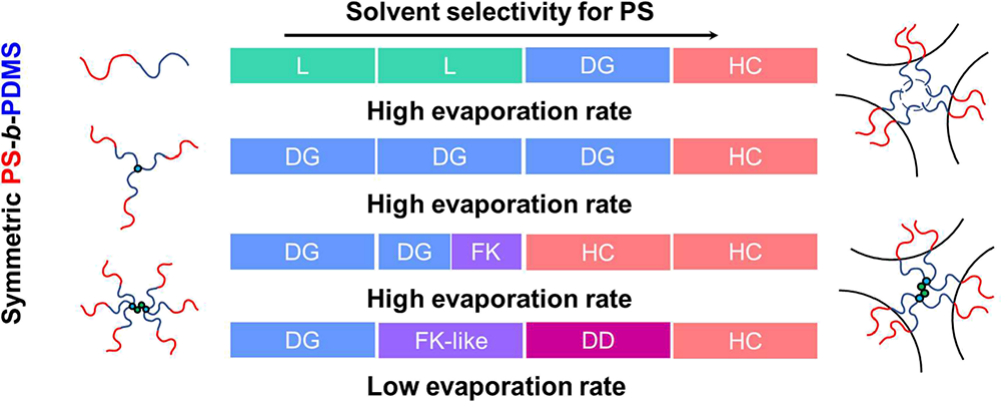
Phase behaviors of diblock and star-block PS-*b*-PDMS by controlled self-assembly.

## Supplementary Material



## Notes

Detailed synthesis route for all samples; NMR and GPC details; SAXS profiles and TEM micrographs after solution casting by cyclohexane; SAXS profiles and TEM micrographs of the controlled self-assembly of diblock, three- and six-arm star BCPs PS-*b*-PDMS; and determination of solvent selectivity to the block copolymers used in this study (PDF)
