# Supplementary material for: Architecture Effect on Network Phase Formation from Controlled Self-Assembly of High‑χ Block Copolymers
Source: Macromolecules. 2025 Nov 17;58(23):12574–81. doi: 10.1021/acs.macromol.5c02152 (PMC12874629; doi:10.1021/acs.macromol.5c02152)
Supplement: Supplementary file 1 [file ma5c02152_si_001.pdf]

Supporting Information

Architecture Effect on Network Phase Formation from Controlled Self-Assembly of High-χ Block Copolymers

Cheng-Yen Chang [a, ‡], *Gkreti-Maria Manesi* [b, ‡], *Yun-Hao Chen* [a], *Yu-Jie Tsai* [a], *Hsing-Yu Su*[a]

Apostolos Avgeropoulos [b,] *, *Rong-Ming Ho* [a,] *

[a] Department of Chemical Engineering, National Tsing Hua University No. 101, Section 2, Kuang-Fu Road, Hsinchu, Taiwan 30013, R.O.C.

[b] Department of Materials Science Engineering, University of Ioannina, University Campus, Ioannina 45110, Greece

‡C.-Y.C, and G.-M.M. contributed equally to this work.

* Apostolos Avgeropoulos
Email: aavger@uoi.gr
orcid.org/0000-0002-6203-9942
*Rong-Ming Ho
Email: rmho@mx.nthu.edu.tw
orcid.org/0000-0002-2429-7617

Characterization of Lamellae-Forming PS-*b*-PDMS

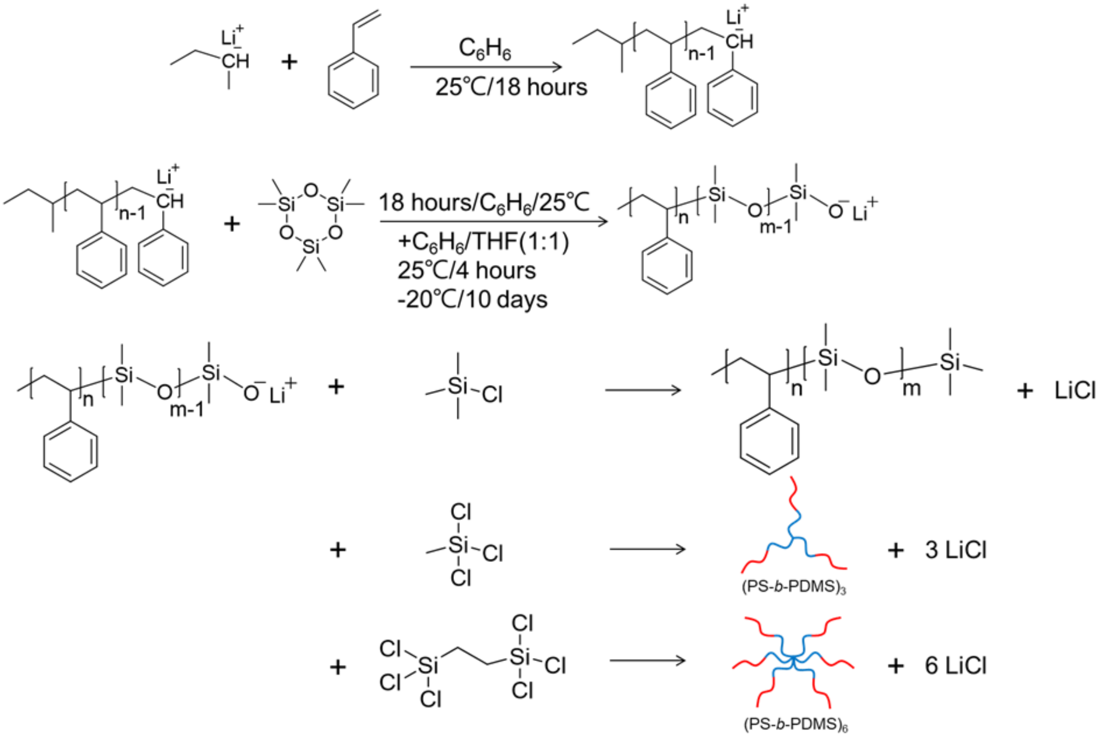

Figure S1. Schematic illustration for synthesis of diblocks, three-arm and six-arm star-blocks PS-*b*-PDMS.

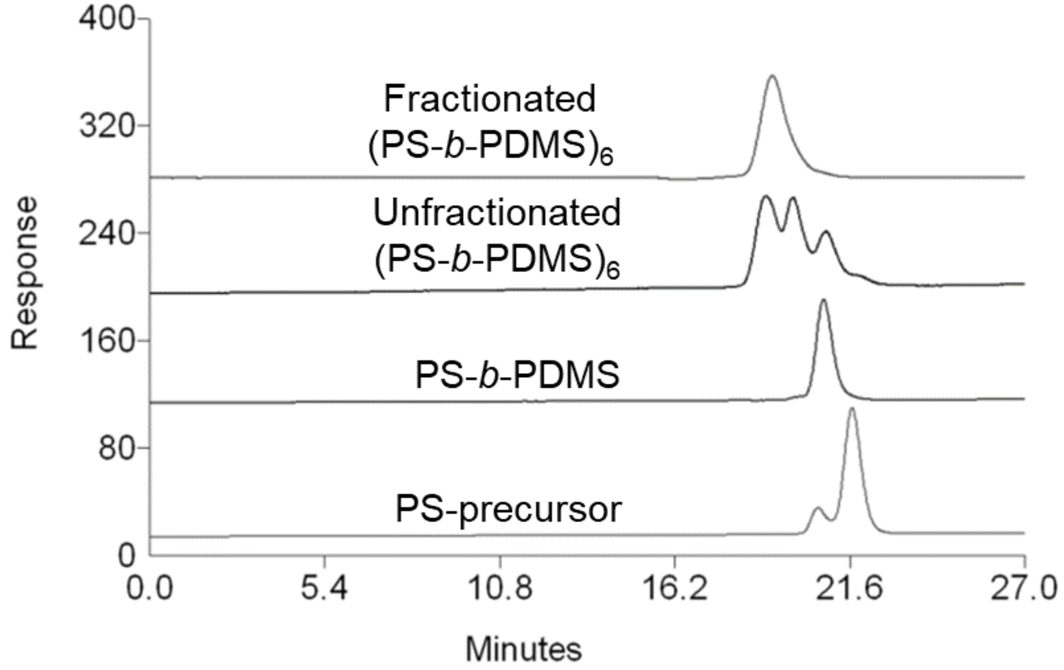

Figure S2. SEC chromatograph of (PS-*b*-PDMS)$_n$, n = 1 or 6. Reproduced from ref 1.

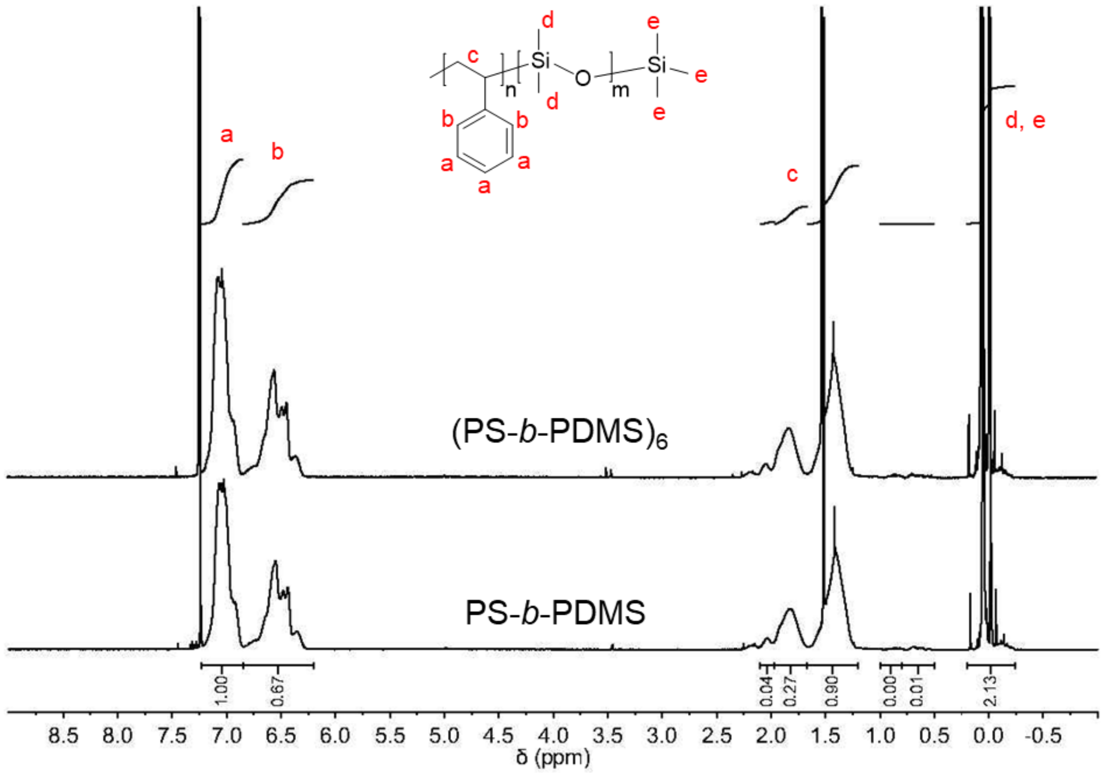

Figure S3. The ^{1}H-NMR spectrum of (PS-*b*-PDMS)$_n$, n = 1 and 6. Reproduced from ref 2.

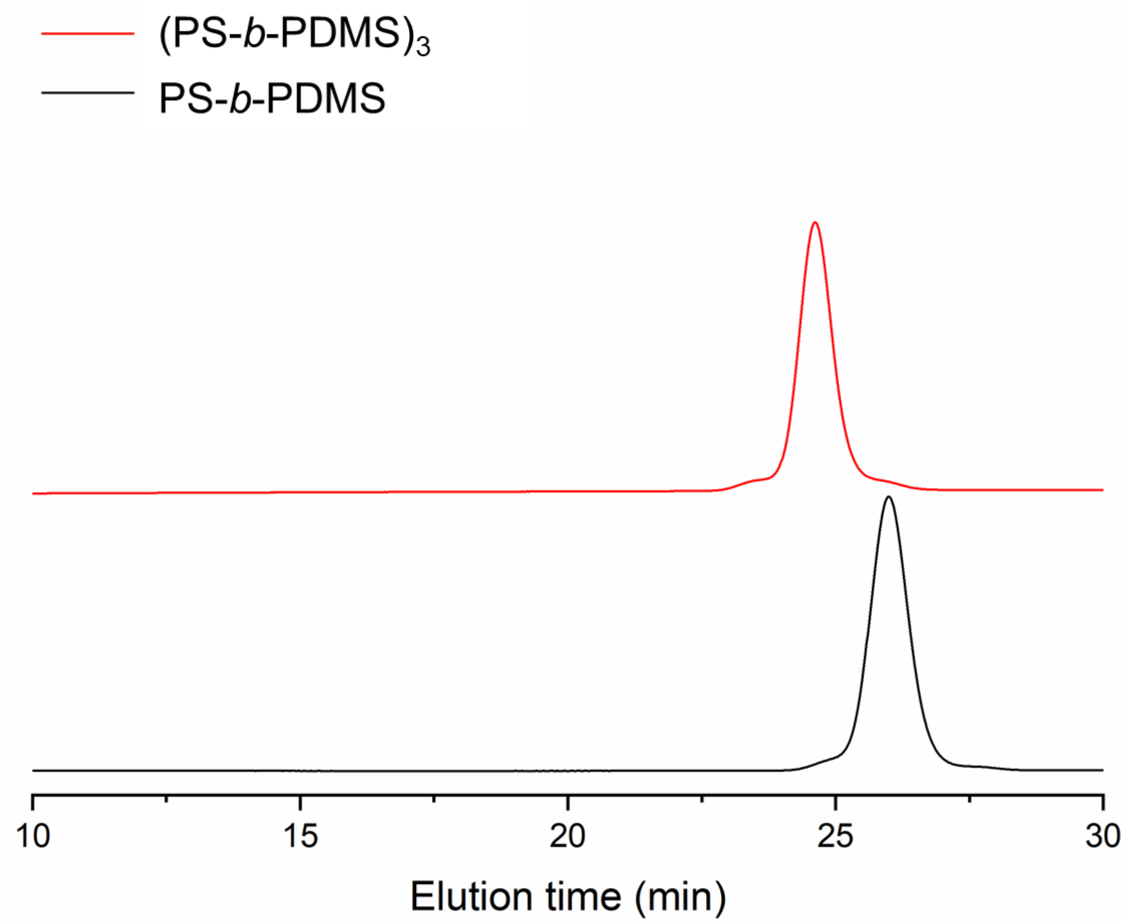

Figure S4. SEC chromatograph of (PS-*b*-PDMS)$_3$ and its diblock precursor.

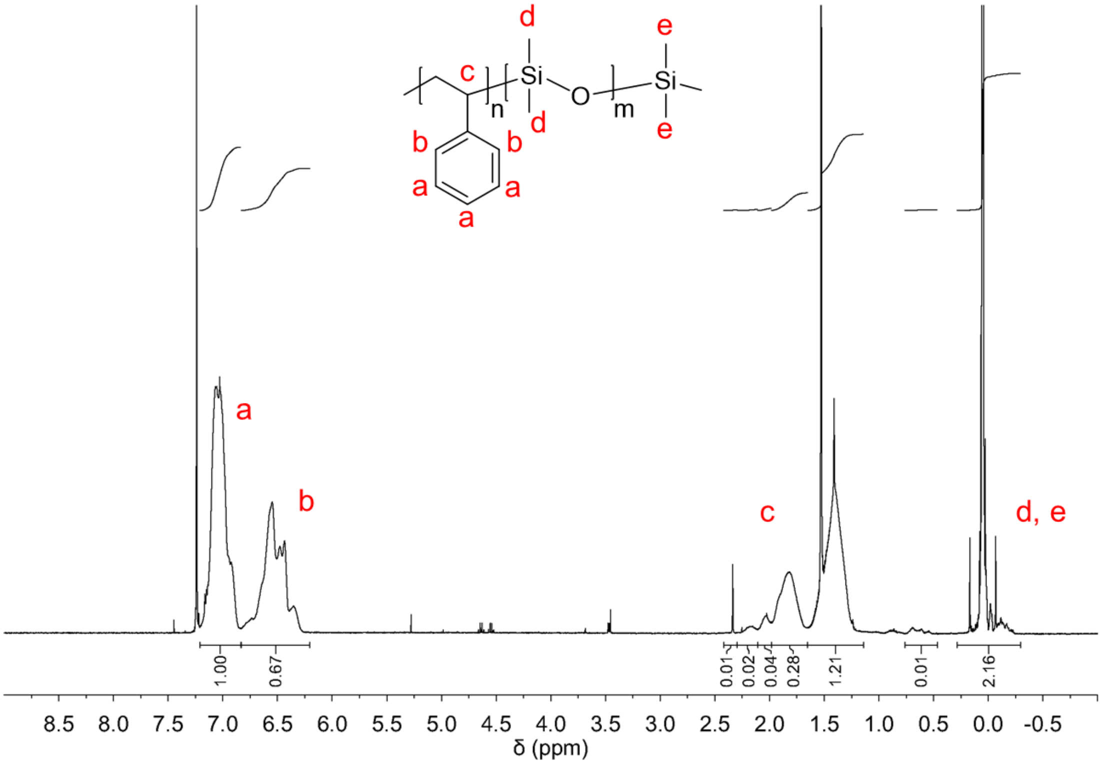

Figure S5. The ^{1}H-NMR spectrum of the diblock precursor of (PS-*b*-PDMS)$_3$.

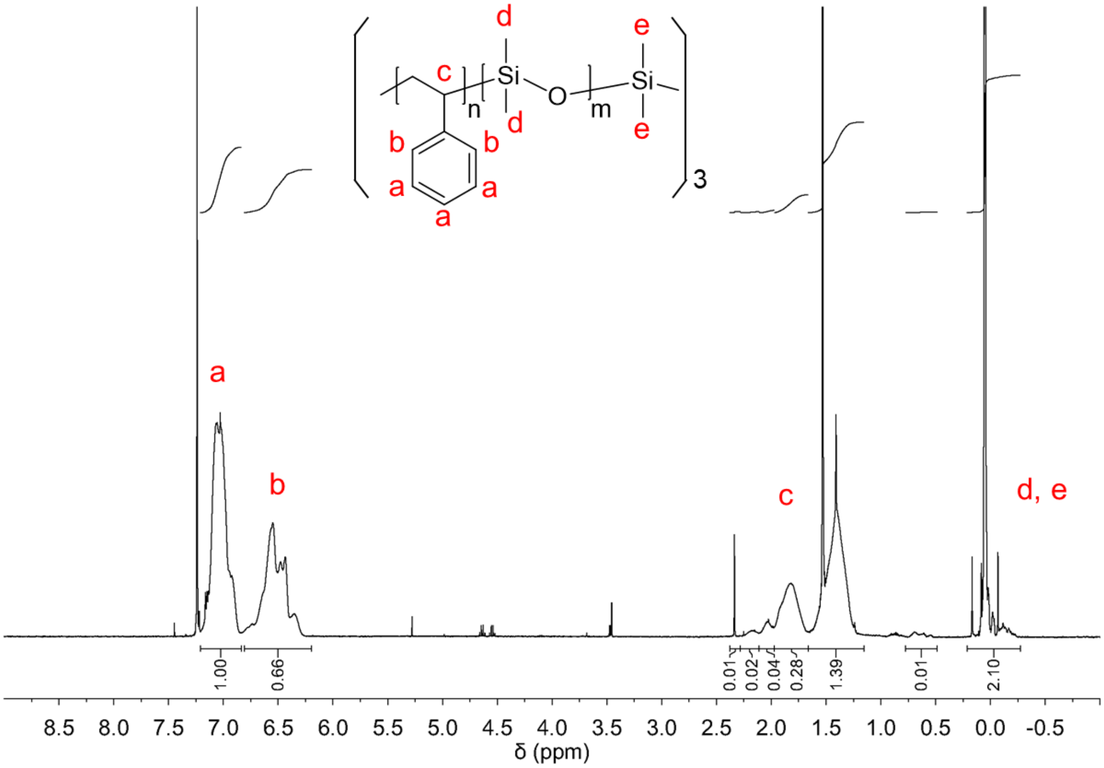

Figure S6. The ^{1}H-NMR spectrum of (PS-*b*-PDMS)$_3$.

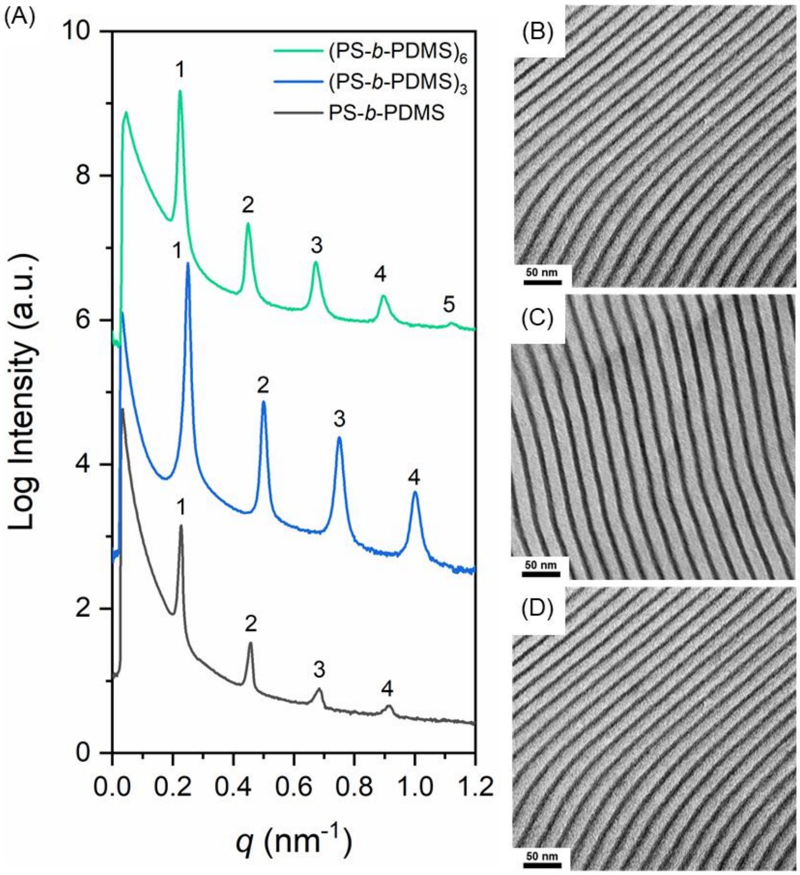

Figure S7. (A) One-dimensional SAXS profiles and TEM micrographs of (B) PS-*b*-PDMS, (C) (PS-*b*-PDMS)$_3$ and (D) (PS-*b*-PDMS)$_6$ from solution casting using cyclohexane.

Determination of Solvent Selectivity to PS-*b*-PDMS

The relative selectivity of solvent toward PS and PDMS were measured by the degrees of swelling ratios of PS and PDMS films under saturated solvent vapor. A synthesized PS homopolymer (Mw: 31,000 g/mol) and a commercialized PDMS homopolymer (Mw: 10,000 g/mol, Polymer Source) were dissolved in cyclohexane as polymer solution at a concentration of 3wt% for preparation of polymer thin films. The thickness of polymer films was fixed to be constant at approximately 150 nm. For the *in-situ* observation of film thickness at swollen state, an interferometer was installed on the sealed chamber where films were stored for solvent annealing as reported in our previous study.(3) The flow rates of solvent vapor were fixed at a constant value of 20 mL/min and hold for 2,500 seconds to record the swelling ratio of polymer films when reaching equilibrium. The relative solvent selectivity to PS and PDMS was therefore determined by the values of swelling ratios of PS divided by that of PDMS under the same environment of solvent vapor and normalized by the vapor pressure of the corresponding solvent during the measurements. The estimation of the effective volume fraction ($f_{\mathrm{PDMS}}^{v,\mathrm{eff}}$) is as follow:

$$f_{\mathrm{PDMS}}^{v,eff} = \frac{\mathrm{V_{PDMS}} \times \mathrm{SR_{PDMS}}}{\mathrm{V_{PDMS}} \times \mathrm{SR_{PDMS}} + \mathrm{V_{PS}} \times \mathrm{SR_{PS}}},$$

$\mathrm{SR_i}$: swelling ratio of component i under saturated vapor pressure of solvent.

To standardize the difference in the vapor pressures of each solvent which cause inconsistent swelling capability, we set the $\mathrm{SR_{PDMS}}$ at 1.16 (the lowest value recorded) and record the $\mathrm{SR_{PS}}$ when $\mathrm{SR_{PDMS}}$ reached the setting point. The $f_{\mathrm{PDMS}}^{v,\mathrm{eff}}$ in the self-assembled PS-*b*-PDMS is therefore estimated in between 0.392 and 0.377 as the selectivity of solvent increases following the sequence of chloroform (0.392), toluene (0.389), DCM (0.380) and chlorobenzene (0.377).

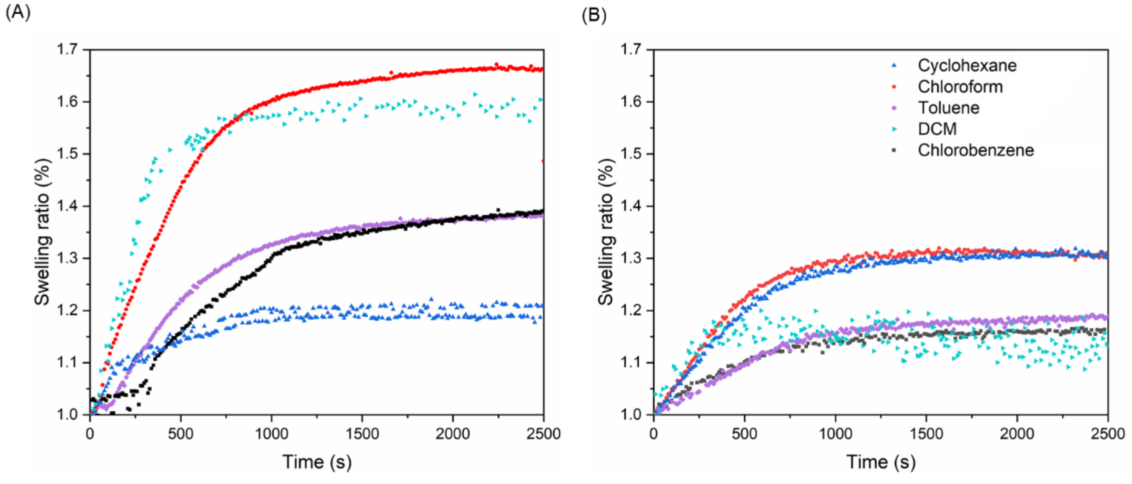

Figure S8. Swelling ratio profile of pure (A) PS and (B) PDMS thin films at an initial thickness of approximately 150 nm during solvent annealing at a fixed flow rate of solvent vapor (20 mL/min).

Phase Behaviors of PS-*b*-PDMS under Slow Evaporation Rate

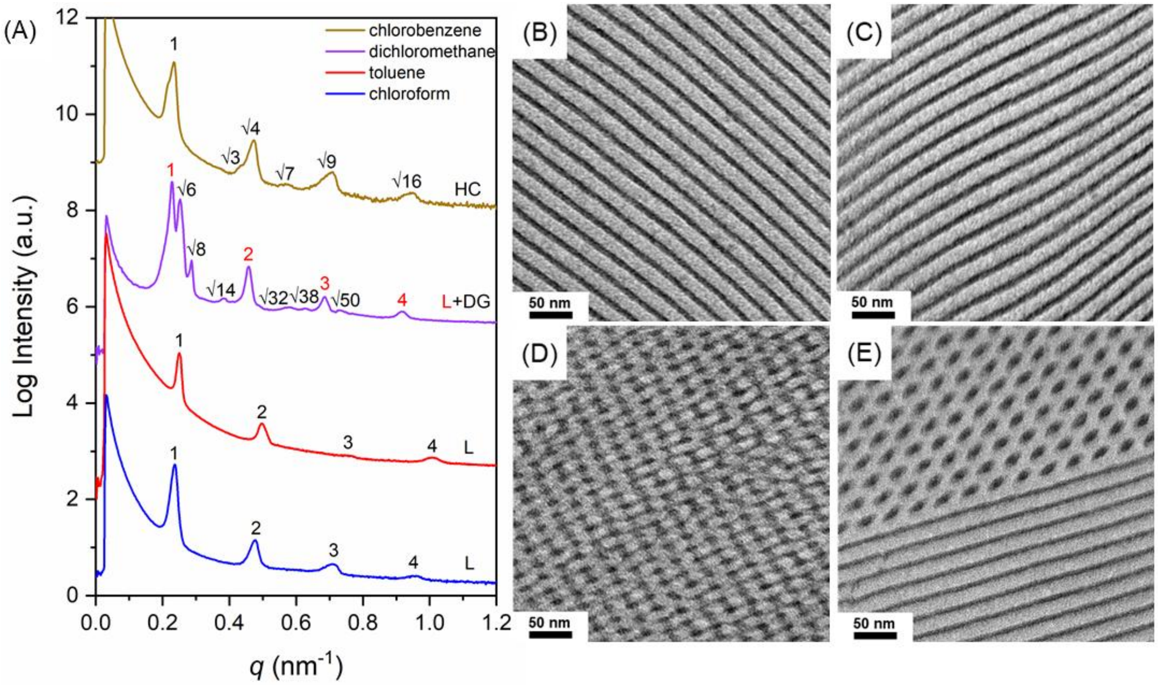

Figure S9. (A) One-dimensional SAXS profiles; (B-E) TEM micrographs of PS-*b*-PDMS from solution casting using chloroform, toluene, dichloromethane and chlorobenzene under slow evaporation rate of 0.1 mL/day. Reproduced from ref 2.

Phase Behaviors of (PS-*b*-PDMS)3 under Slow Evaporation Rate

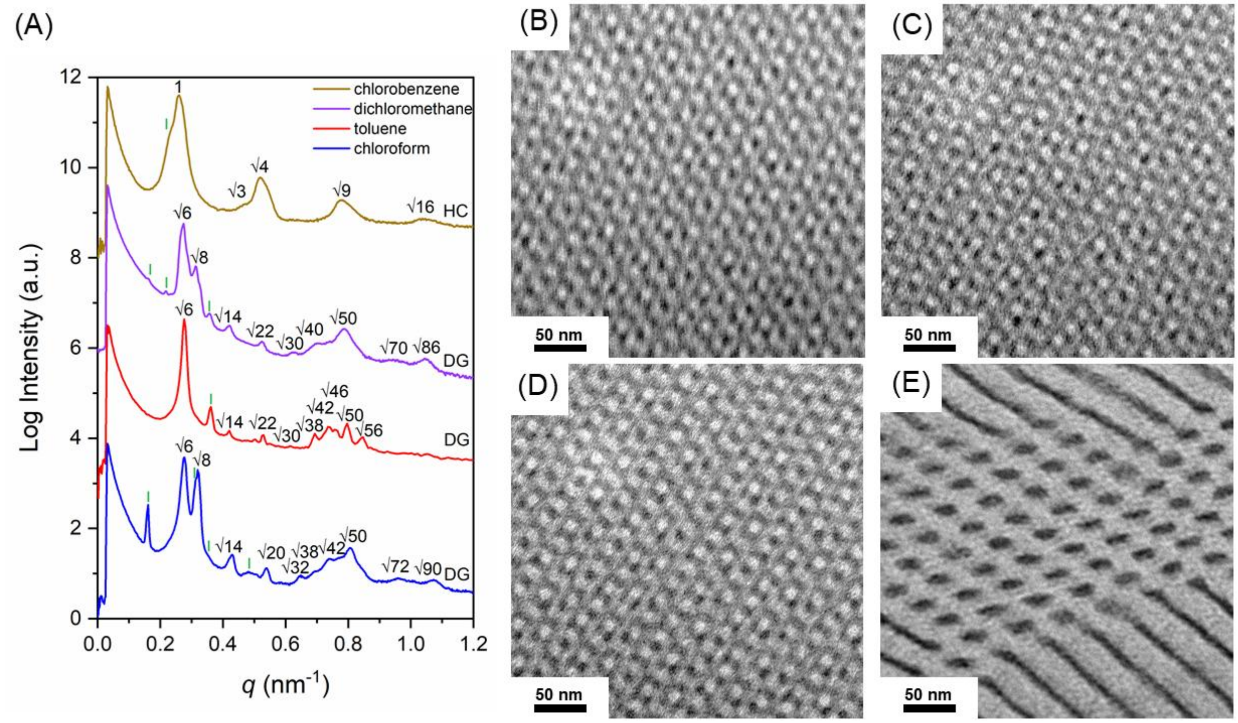

Figure S10. One-dimensional SAXS profiles of (A) (PS-*b*-PDMS)3; TEM micrographs of (B-E) (PS-*b*-PDMS)3 from solution casting using chloroform, toluene, dichloromethane and chlorobenzene under slow evaporation rate of 0.1 mL/day.

Phase Behaviors of (PS-*b*-PDMS)$_6$ under Slow Evaporation Rate

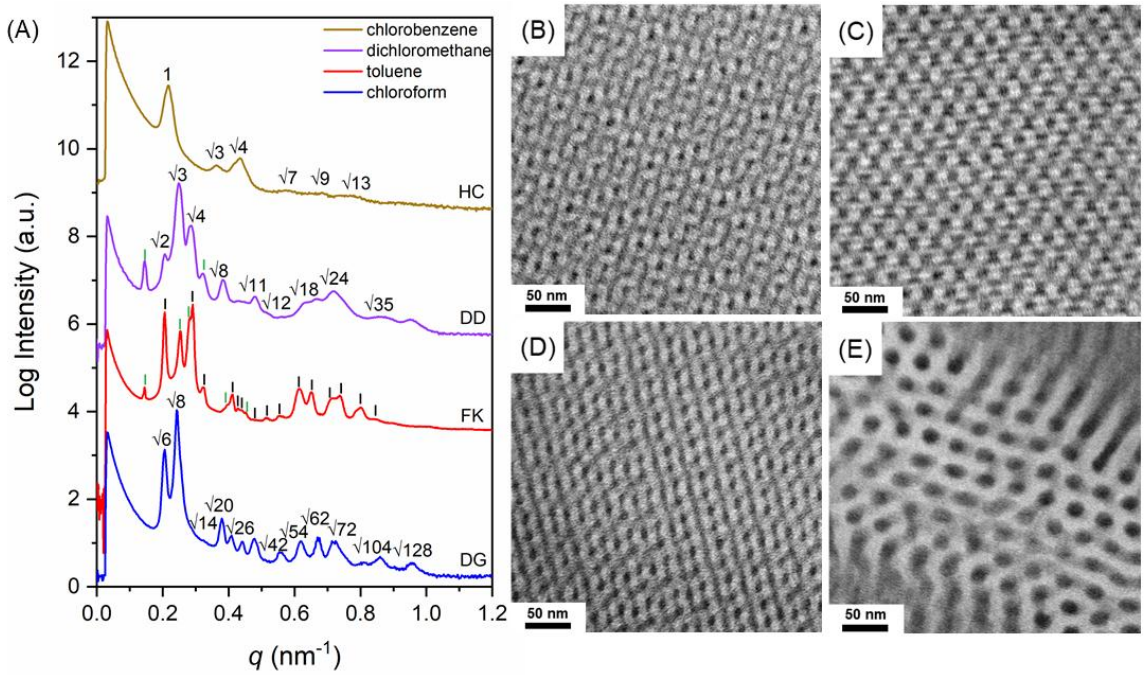

Figure S11. (A) One-dimensional SAXS profiles; (B-E) TEM micrographs of (PS-*b*-PDMS)$_6$ from solution casting using chloroform, toluene, dichloromethane and chlorobenzene under slow evaporation rate of 0.1 mL/day. Reproduced from ref 2.

SI References

1. G.-M. Manesi, C.-Y. Chang, I. Moutsios, R.-M. Ho, A. Avgeropoulos, Tuning the morphology of silicon containing copolymers via macromolecular architecture effect. *Giant* **16**, 100190 (2023).
2. C. Y. Chang *et al.*, Frank-Kasper-like network phase from self-assembly of high-χ star-block copolymers. *Sci Adv* **10**, eado4786 (2024).
3. Y. H. Wu, T. Y. Lo, M. S. She, R. M. Ho, Morphological Evolution of Gyroid-Forming Block Copolymer Thin Films with Varying Solvent Evaporation Rate. *ACS Appl Mater Interfaces* **7**, 16536-16547 (2015).
